# Disentangling the Drivers of Fish Community Variation in a Highly Urbanized Estuary Based on Hierarchical Modeling of Species Communities (HMSC)

**DOI:** 10.1002/ece3.74523

**Published:** 2026-10-26

**Authors:** Longjie Zhang, Wencheng Zhao, Shuai Zhang, Shiyi Li, Min Li, Keshu Zou

**Affiliations:** ^1^ College of Marine Sciences South China Agricultural University Guangzhou China; ^2^ Guangdong Provincial Observation and Research Station for Ecosystem in the Pearl River Estuary, South China Sea Fisheries Research Institute Chinese Academy of Fishery Sciences Guangzhou China

**Keywords:** environmental filtering, fish community, functional traits, hierarchical modeling of species communities, Pearl River Estuary

## Abstract

Biodiversity conservation and management in highly urbanized estuaries have garnered widespread global attention. However, the environmental response mechanisms of fish communities in these estuaries remain poorly understood, hindering their ecological health and sustainable development. Here, we utilized Hierarchical Modeling of Species Communities (HMSC), a burgeoning and powerful framework, jointly investigating how the species occurrences, species traits, and spatiotemporal structure of fish communities respond to environmental variables in the Pearl River Estuary (PRE). It exhibited seasonal variation in species composition, taxonomic diversity, functional diversity, and functional‐space occupancy of fish communities, and several CWM traits showed directional seasonal shifts. Furthermore, the HMSC framework showed that environmental filtering was the primary driver of the spatiotemporal dynamics in fish community. Salinity, pH, and phosphate were identified as key environmental drivers of species distributions and trait‐mediated responses. Functional traits showed contrasting responses along the phosphate gradient, whereas habitat‐level residual species associations revealed distinct positive and negative co‐occurrence patterns that reflect shared environmental filtering rather than stochastic assembly. The study elucidates the environmental response mechanisms of fish community dynamics by integrating taxonomic and functional diversity perspectives, offering new insights into the assembly of estuarine fish communities.

## Graphical abstract

Estuarine fish communities in highly urbanized areas face complex environmental pressures. Using a powerful Hierarchical Modeling of Species Communities (HMSC) framework, this study reveals that salinity, pH, and phosphate levels act as key environmental filters driving the seasonal dynamics of fish taxonomic and functional diversity in the Pearl River Estuary. Our findings provide crucial insights for the ecological management and conservation of urbanized estuarine ecosystems.

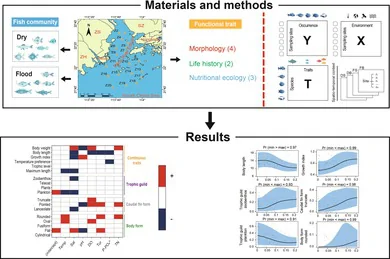

## 1 Introduction

Estuaries are shaped by complex processes, including water mixing, material exchange, biological interactions, and tidal pulses. These processes create highly dynamic environmental conditions and support abundant biological resources (Yu et al. 2023). It provides ideal habitat conditions for endangered species, as well as species of significant ecological and economic importance, which are crucial for sustaining regional ecosystem stability and supporting local economic development. As the fourth largest estuarine region in the world, the Pearl River Estuary (PRE) in China is distinguished by its pronounced geographic complexity and a highly interconnected fluvial network, which discharges into the northern South China Sea through eight distributaries (Hou et al. 2021). It receives substantial annual rainfall, which contributes to high levels of primary productivity. Collectively, these hydrological and ecological characteristics render it a nationally significant fishery habitat (Kuang et al. 2021; Li, Jiang, et al. 2023). However, estuarine ecosystems are among the most vulnerable ecological environments on the planet (Peng et al. 2021). The region's abundant aquatic resources and strategically significant geographical location have, over time, led to growing fishing pressures and other anthropogenic activities in the PRE (Liu et al. 2024). These cumulative pressures have ultimately resulted in the depletion of fishery stocks and exacerbated environmental degradation, consequently precipitating a decline in biodiversity and compromising ecosystem stability (Li, Jiang, et al. 2023; Mai et al. 2023).

Elucidating the functional diversity and assembly mechanisms of biological communities is crucial for advancing biodiversity conservation and promoting sustainable development in estuarine ecosystems (Li, Jiang, et al. 2023). Fish, as key biological communities of aquatic ecosystems, play critical roles in energy flow and material cycling. Understanding the mechanisms underlying fish community dynamics has become a central focus of regional aquatic ecosystem research. Previous studies have shown that fish community formation is influenced by a wide range of environmental changes and anthropogenic disturbances (Danet et al. 2024). As an integral component of estuarine ecosystems, fish communities are highly susceptible to various stressors, including environmental changes, fishing pressure, and stochastic disturbances, all of which contribute to the decline of fish species diversity (Zeng et al. 2019). Moreover, functional traits of fish have been demonstrated to be sensitive to environmental changes during ecosystem disturbances (Brandão et al. 2025; Lai et al. 2024). Compared with species‐based approaches, trait‐based studies offer more profound insights into the relationships between fish communities and environmental factors, thereby improving our understanding of the mechanisms underlying fish community responses to environmental change (Baptista et al. 2021; Coleman et al. 2015; McLean, Mouillot, Lindegren, et al. 2019). It is increasingly acknowledged that higher functional diversity within fish communities can significantly enhance their resilience to extreme environmental conditions (Costa et al. 2024; Zhang et al. 2025). Despite advancements in comprehending the impacts of environmental fluctuations on fish life history, nutrient composition, and phenotypic orientation, the relationship between the environmental variation of fish functional traits remains unclear (Cheng et al. 2023; Lai et al. 2022; Li, Wang, et al. 2023; Zhu et al. 2024). Enhancing our understanding of formation mechanisms in fish functional diversity can facilitate a more comprehensive grasp of their ecological adaptability and functionality (Chang et al. 2024).

There is no doubt that the establishment and persistence of species in local communities primarily depend on their environmental and resource requirements, a process commonly referred to as environmental filtering (Ford and Roberts 2020; Murray‐Stoker et al. 2022; Portela et al. 2023). Recent studies have proved that species' functional traits and biotic interactions can differentially mediate their responses to environmental filtering (Cordero and Jackson 2023; Mellin et al. 2016; Yeager and Hughes 2025). Accordingly, we hypothesize that environmental filtering integrates not only species' functional traits but also biotic interactions and other ecological factors (Bauer et al. 2022; Cantera et al. 2023; Lopez and Freestone 2022). Moreover, the influence of environmental filtering on fish communities is constrained by scale‐dependent properties of ecological systems, particularly spatial and temporal dimensions (Chen et al. 2022; Zbinden 2020). Some scholars have therefore proposed that environmental filtering as a hierarchical process, in which the effects of sampling time and location must be considered (O'Mara et al. 2024; Ramírez‐García et al. 2022). By incorporating spatio‐temporal contexts into the study of environmental filtering, ecological inferences about community assembly processes can be drawn more robustly (Ovaskainen and Abrego 2020).

Recently, Joint Species Distribution Models (JSDMs) have been increasingly utilized due to their capacity to quantify the effects of environmental variables on species abundances at the community level, as well as their superior capacity to disentangle multiple processes underlying community assembly (Franklin 2023; Ovaskainen et al. 2017). As one of the most advanced models among the JSDMs, Hierarchical Model of Species Community (HMSC) facilitates a more comprehensive analysis of multi‐level hierarchical structures and spatiotemporal data configurations by integrating various data types, such as environmental covariates, species phylogenetic associations, and relevant life‐history traits (Murillo et al. 2020; Ovaskainen and Abrego 2020; Tikhonov et al. 2020). It employs generalized linear (mixed) regression within a hierarchical Bayesian framework to characterize species‐ and trait‐level responses to environmental drivers (Abrego et al. 2017). Additionally, random effects are examined in the HMSC to assess potential interspecific interactions. The HMSC framework has been successfully applied to studying the drivers for fish community assembly in various aquatic ecosystems, such as river ecosystems in the Amazon region, the bottom ecosystem of the North Sea, and coral reef ecosystems in the Atlantic Ocean (Gomes et al. 2023; Keppeler et al. 2022; Montanyès et al. 2023; Xu et al. 2025). Nevertheless, the application of HMSC in analyzing the changes of fish communities within estuarine ecosystems remains relatively limited, especially the application to investigate seasonal or quarterly dynamics of fish communities in estuarine environments.

In this study, the dynamics of fish community in the PRE from both taxonomic and functional diversity perspectives were investigated. Then HMSC framework was then employed to jointly model species occurrence data, environmental variables, functional traits, and spatiotemporal structure, thereby elucidating how fish communities respond to environmental change in the PRE. The main objectives of this study are to: (1) explore the seasonal variations of fish communities and the underlying driving factors for the dynamics in the PRE; (2) evaluate and quantify how specific fish species respond to specific environmental factors; and (3) assess the role of species functional traits in fish community responses to key environmental drivers. It is anticipated to yield valuable insights and establish scientific foundations for the sustainable development of estuarine biodiversity and ecosystems.

## 2 Materials and Methods

### 2.1 Study Area and Sampling

A total of 20 sampling sites in the PRE were surveyed during the dry (February) and flood (September) seasons of 2023 (Figure 1A). Conventional bottom trawling was conducted at each site to collect fish specimens. A single bottom trawl with a 36‐m headline, a total net length of 50 m, a 5‐cm mesh size at the net opening, and a 3‐cm cod‐end mesh size was used. Each tow lasted 0.5 h, and the trawling depth corresponded to the local bottom depth at each sampling site. The identification and classification processes were based on Fishbase (Froese and Pauly 2025), Atlas Guide to Economic Fishes of South China Sea (Yan et al. 2021), and Fishes of the Pearl River (Li 2018). Based on habitat information obtained from FishBase and the relevant literature, each species was assigned a priori to either a pelagic‐associated or demersal‐associated habitat group. The catches assigned to each group were then summarized separately for each station and season (Gao et al. 2024). Considering the seasonal variations of the samples, a total of four fish communities were obtained, namely the surface (DS)/bottom (DB) layer in the dry season, and the surface (FS)/bottom (FB) layer during the flood season.

**FIGURE 1:**
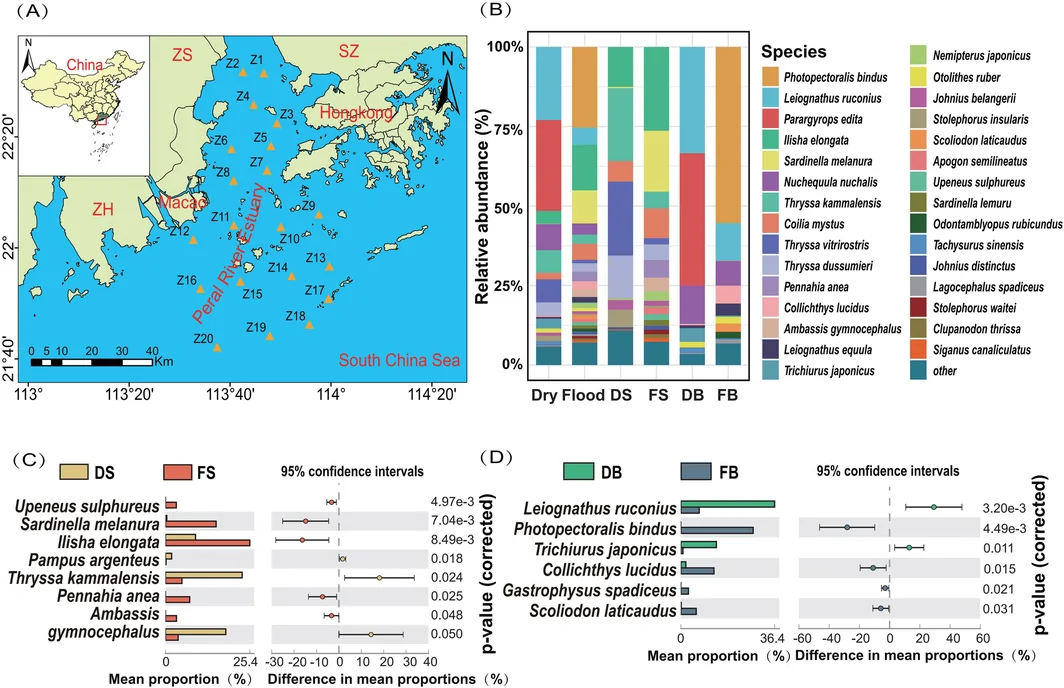
Sampling sites, composition and differences of fish communities in the Pearl River Estuary (PRE). (A) The detailed information of the studying area including the approximate location of the PRE in southern China and the sampling points. Black triangles indicate the specific location distribution of each sampling point. ZH/ZS/SZ represent Zhuhai City, Zhongshan City, and Shenzhen City, respectively. (B) Relative abundance of fish communities composition in different groups, showing the top 30 species. (C) Significant differences in species relative abundance between dry‐ and flood‐season surface fish communities, identified using STAMP. (D) Significant differences in species relative abundance between dry‐ and flood‐season bottom fish communities, identified using STAMP. In panels (C) and (D), horizontal bars show mean relative abundances, and points with error bars indicate differences in mean proportions and their 95% confidence intervals; corrected *p* values are shown on the right. Dry/Flood refers to fish community in the dry/flood season, DS/DB represents the surface/bottom fish community in the dry season, and FS/FB represents surface/bottom fish community in the flood season.

At each sampling site, the longitude (lon) and latitude (lat) of each sampling site were recorded using a GPS measuring instrument (Magellan, USA). Environmental factors, including dissolved oxygen (DO, mg/L), pH, salinity (Sal, ppt), and water temperature (Temp, °C), were measured in situ using an YSI multi‐probe water quality meter (YSI Inc., OH, USA). Water current velocity (CV, m/s) and turbidity (Tur, NTU) were monitored through the Velocity meter (LS1206B, Nanjing Xiangruide Electric Technology Co. Ltd., China) and the turbidity meter (TD‐M500, Optex Co. Ltd., Japan). A 5‐L water sample in each sampling site was store at 4°C and transported back to the laboratory within 24 h for follow‐up experiments. The following detected environmental factors were: total nitrogen (TN), nitrite (N–NO_2_^−^), nitrate (N–NO_3_^−^), ammonia nitrogen (N–NH_4_^+^), and phosphate (P–PO_4_^3−^) following methods outlined in previous studies (Ma et al. 2025; Sun et al. 2016; Xie et al. 2016).

### 2.2 Functional Traits Selection

Nine functional traits were selected in our study, including body form, caudal fin form, maximum length, trophic guild, trophic level, temperature preference, growth index, body length, and body weight. The selection of functional traits was based on four major categories: life history, habitat preference, trophic ecology, and locomotion, which are commonly used in trait‐based studies of fish communities (for a list of traits with references, see Table S1). These categories had been validated as relevant for investigating fish community assembly and functional diversity (Ladds et al. 2018; McKinley et al. 2022; Villeger et al. 2010). A fundamental assumption underlying the use of a single trait estimation was that trait values were invariant, remaining constant across time and space for a given species, as well as throughout individual development (Zhao et al. 2022). The data of functional traits for the majority of species were obtained from FishBase (Froese and Pauly 2025) through the “rfishbase” R package (Boettiger et al. 2012). For the missing data of species traits, the values were carefully reviewed and supplemented using relevant literature and reference books (Dumay et al. 2004; Elliott et al. 2007; Feng et al. 2023; Jia et al. 2022; Rincón‐Díaz et al. 2018; Trindade‐Santos et al. 2022; Villéger et al. 2017).

### 2.3 Data Analysis

The compositions of fish communities in different groups were visualized using a bar graph. Seasonal differences of fish communities were further assessed using STAMP (Statistical Analysis of Metagenomic Profiles, v2.1.3; Parks et al. 2014). Species relative‐abundance profiles were compared between the dry and flood seasons separately for the surface‐associated and bottom‐associated groups using Welch's *t*‐test. Differences in mean proportions were estimated together with 95% confidence intervals, and species with corrected *p* values < 0.05 were considered significantly different. Diversity indices of fish community, encompassing both taxonomic and functional levels, were calculated, and statistical significance was assessed through *t*‐tests using the “vegan” and “mFD” packages (Magneville et al. 2022; Oksanen et al. 2025). Additionally, nonmetric multidimensional scaling (NMDS) based on Bray–Curtis dissimilarity was used to visualize differences in overall fish communities between seasons and ecological groups. ANOSIM was further applied to test whether community structure differed significantly between the dry and flood seasons, as well as between DS and FS and between DB and FB (Qian et al. 2023).

The “FD” package was used to calculate community‐weighted means (CWMs) for dominant fish functional traits (Laliberte and Legendre 2010). Seasonal differences in continuous CWM traits were evaluated separately for surface‐ and bottom‐associated communities using paired permutation tests with 9999 permutations, whereas seasonal shifts in categorical CWM traits were assessed using Stuart‐Maxwell tests of marginal homogeneity. *p* values were adjusted for multiple comparisons using the Benjamini‐Hochberg procedure, with adjusted *p* < 0.05 considered statistically significant (Zhu et al. 2026). The multidimensional traits space of fish communities in the PRE was created by applying Principal Coordinate Analysis (PCoA) of the Gower dissimilarity matrix based on functional traits using the “mFD” package (Magneville et al. 2022). The functional convex hulls were plotted to represent fish communities based on species composition, and the quality of the functional space was evaluated using the “quality.fspaces.plot” function from the mFD package (Lai et al. 2022). The correlations between specific trait axes and functional axes were examined, utilizing the multidimensional average deviation (MAD) as a measure of robustness in functional space, and a smaller value could lead to a more stable functional space (Magneville et al. 2022). The seven‐dimensional space was selected for constructing the functional space, as it exhibited the highest quality. It could also be evidenced by the lowest MAD, ranging from 0.034 to 0.039 (Table S2). Then, the first four axes were utilized to represent the functional space in order to facilitate more effective visualization of the graph, collectively accounting for 50.8%–60.1% of the explained variance (Table S2). Additionally, we calculated six functional diversity indices, including functional richness (FRic), functional divergence (FDiv), functional specialization (FSpe), functional originality (FOri), functional mean pairwise distance (FMPD), and functional mean nearest neighbor distance (FNND), and the difference in the functional composition between fish communities was compared based on species' positions in a seven‐dimensional functional trait space and their relative abundances.

Simultaneously, the presence of multicollinearity among environmental factors was evaluated through variance inflation factor (VIF) analysis, and highly correlated predictors were excluded to ensure the robustness of the modeling. Moreover, given the influence of the site's geographic coordinates (lat and lon) on the process of community assembly, these parameters were incorporated as similar as environmental factors into the dataset (Ford and Roberts 2020).

### 2.4 Construction of the Hierarchical Model for Species Communities (HMSC)

The capabilities of HMSC included analyzing species responses to environmental changes, quantifying contributions of deterministic and stochastic processes, assessing species interactions across spatial scales, forecasting species or functional traits distribution patterns along environmental gradients (Abrego et al. 2017; Pollock et al. 2014). Preliminary abundance‐based HMSC models showed poor MCMC convergence (PSRF > 1.1), we converted abundance to presence‐absence data and fitted a probit HMSC model integrating seasons and habitats (Tikhonov et al. 2020; Lin et al. 2025). It also aligned with our primary objective of assessing environmental filtering and species‐environment relationships. A presence‐absence HMSC model with a probit link function was fitted using individual hauls as sampling units. Bayesian inference was performed via Markov Chain Monte Carlo (MCMC) sampling to estimate species‐level and trait‐level responses to fixed effects (i.e., environmental variables and biotic interactions) and random effects (i.e., spatial and temporal structures). In order to prevent the model from overfitting, further collinearity screening of environmental factors was conducted, and finally seven environmental factors (i.e., temperature, salinity, pH, dissolved oxygen, turbidity, phosphate, and total nitrogen) were included as fixed effects. Species data were transformed into presence‐absence format and used as the response variable, while species‐level trait data were included as explanatory variable. Besides, three random effects were incorporated: sampling site and water layer as spatially explicit effects, and the sampling seasonal within year (dry and flood seasons) as unstructured temporal effects (Montanyès et al. 2023). The above three random effects were modeled independently and corresponded to sample sites, habitat and season, respectively. The maximum number of latent factors per random effect was set to 10. In addition, species occurring at fewer than five sampling sites across the entire dataset were excluded to reduce instability in parameter estimation for extremely sparse occurrence data (Montanyès et al. 2023). Based on this prevalence criterion, 84 of the 139 recorded species were excluded, leaving 55 species for the final HMSC analysis. None of the excluded species was identified as a rare endemic species or a species of particular ecological importance.

Following model specification, we fitted the model using four MCMC chains with 1000 posterior samples retained per chain. During preliminary tuning, thinning intervals of 1, 10, 100, and 1000 were evaluated based on PSRF and ESS, and a thinning interval of 1000 was selected for the final model to ensure satisfactory convergence. A transient period of 50,000 iterations was discarded, resulting in 1,050,000 iterations per chain and 4000 retained posterior samples across the four chains. Convergence was considered satisfactory when the maximum PSRF for each monitored parameter group was below 1.1 (Tikhonov et al. 2020). Then, we quantified and visualized species‐specific responses to environmental factors (beta) and trait‐mediated variation in species‐environment relationships (gamma) at the community level. Residual species associations (omega), derived after accounting for shared environmental responses, were estimated for the random‐effect levels of sampling site, season, and habitat. Parameter estimates with posterior probabilities greater than 0.9 were considered statistically well supported (Tikhonov et al. 2020). Furthermore, we evaluated two trait‐related variance components to assess the role of functional traits in mediating community assembly processes. The first component (*R* ^2^_TY_) represents the proportion of variance in species occurrences explained by functional traits, whereas the second component (*R* ^2^_Tbeta_) quantifies the proportion of variance in species‐environment relationships explained by traits (Ovaskainen and Abrego 2020). Finally, model predictive performance was evaluated using fourfold cross‐validation. Specifically, the mean values of the area under the receiver operating characteristic curve (AUC), Tjur *R* ^2^, and root mean square error (RMSE) were calculated. The metrics AUC, Tjur *R* ^2^, and RMSE were used to evaluate the model's discriminatory power and predictive accuracy, respectively (Tjur 2009). AUC measured the discriminatory capacity of species distribution models, with values of 0.5 indicating random performance, 0.7–0.8 considered acceptable, 0.8–0.9 excellent, and values above 0.9 outstanding (Mandrekar 2010). Tjur *R* ^2^ and RMSE were employed to assess the predictive performance of the model. Tjur *R* ^2^ represented the proportion of variation in species occurrence explained by the model, higher Tjur *R* ^2^ indicated higher explanatory accuracy. RMSE reflected the average deviation between predicted and observed values; lower RMSE indicated higher predictive accuracy. All the data analyses related to the HMSC framework were conducted by using the “Hmsc” package in the R software (version 4.4.3).

## 3 Result

### 3.1 Composition and Diversity of Fish Community at Taxonomic Level

A total of 38,248 fish were captured at the two seasons, including 139 species belonging to 2 classes, 19 orders, 56 families, and 96 genera. The three most heavily represented orders were Clupeiformes, Acanthuriformes, and Perciformes, with significant differences between different groups. Clupeiformes (52%) and Acanthuriformes (46%) dominated in the DS, whereas Perciformes (57%) and Acanthuriformes (34%) were predominant in the DB. In contrast, the FS was dominated by Clupeiformes (74%) and Perciformes (22%), whereas Acanthuriformes (71%) and Perciformes (21%) were the dominant taxa in the FB. The composition of fish community in each group was shown at the species level in Figure 1B, with the highest number of species in FB (58), followed by FS (53), DB (43), and DS (42). The six dominant species were *Photopectoralis bindus, Leiognathus ruconius, Paerargyrops edita, Ilisha elongata, Sardinella melanura*, and *Leiognathus nuchalis* . And the Stamp analysis unveiled notable disparities in fish community composition across different seasons (Figure 1C). In the surface layer, highly significant differences (*p* < 0.001) between the two seasons were observed for *Upeneus sulphureus* , *Ilisha elongata* , and *Sardinella melanura* , all of which were exclusively found in the dry season. In the bottom layer, *Trichiurus japonicus, Photopectoralis bindus*, and *Leiognathus ruconius* showed highly significant differences (*p* < 0.001). The *Photopectoralis bindus* was exclusively found in the flood season, whereas the other two species were only present in the dry season. Additionally, the α‐diversity indices of Richness (*p* < 0.001), Shannon (*p* < 0.05), Simpson (*p* < 0.05), and inverse Simpson (*p* < 0.05) showed statistically significant seasonal heterogeneity of fish community in PRE (Figure S1A). NMDS and ANOSIM revealed significant differences in fish community composition between the dry and flood seasons (*R* = 0.344, *p* = 0.001), as well as between DS and FS (*R* = 0.331, *p* = 0.001) and between DB and FB (*R* = 0.252, *p* = 0.001) (Figure S1B).

### 3.2 Composition and Diversity of Functional Traits

Seasonal variation in CWM traits was observed in fish communities of the PRE, particularly for maximum length, trophic guild, body weight, temperature preference, and body length (Figure 2A–E). During the dry season, plankton‐feeding trophic guilds predominated in both DS and DB communities, accounting for 77.5% of sampling sites, whereas invertebrate‐feeding guilds became more prevalent (35%) during the flood season (Figure 2B). Short maximum length and ultralight body weight were also dominant during the dry season (both 52.5%), while medium maximum length (27.5%) and medium body weight (30%) became more prevalent during the flood season (Figure 2A–C). Similarly, thermophilic temperature preference (67.5%) and short body length (55%) were dominant during the dry season, but their proportions declined to 27.5% and 30%, respectively, during the flood season, accompanied by an increased prevalence of medium body length (40%) (Figure 2D,E). Seasonal variation in body form, caudal fin form, trophic level, and growth index *K* was comparatively less pronounced (Figure 2F–I). Besides, growth index *K* and body length were higher and temperature preference was lower in the flood season, while trophic‐guild composition differed between seasons in both surface and bottom‐associated communities. However, neither the paired permutation tests for continuous traits nor the Stuart–Maxwell tests for categorical traits detected significant seasonal differences after Benjamini–Hochberg correction (adjusted *p* > 0.05; Table S3).

**FIGURE 2:**
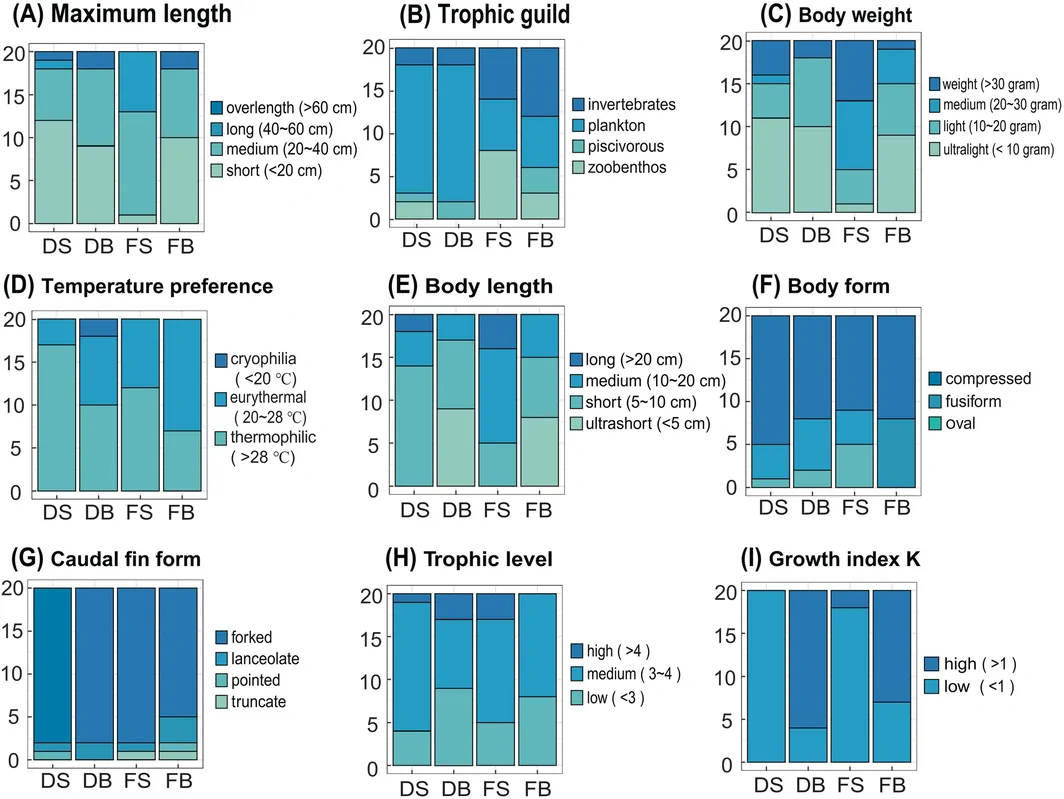
Differences in Community‐weighted mean (CWM) trait values among different communities: (A) Maximum length; (B) Trophic guild; (C) Body weight; (D) Temperature preference; (E) Body length; (F) Body form; (G) Caudal fin form; (H) Trophic level; (I) Growth index *k*. The numbers on the vertical axis indicate the number of sampling points.

The ordination analysis of functional space revealed that four axes accounted for 56.2% (PCoA1: 23.4%, PCoA2: 15.4%, PCoA3: 10.2%, and PCoA4: 7.2%) of the total variability. According to functional space occupancy, the highest proportion (82.24%) was in FB, followed by FS (80.29%). The fish community of DS (71.71%) and DB (65.34%) showed lower levels of space occupancy, respectively (Figure 3). According to the result of correlation between traits and functional axes (Table S4), the first axis (PCoA1) in the functional space was mainly correlated with body length (the correlation coefficient was 0.526), maximum length (0.404), caudal fin form (0.343), and growth index *K* (0.313). The second axis (PCoA2) was mainly correlated with body form (0.507) and caudal fin form (0.379). The third axis (PCoA3) was mainly correlated with body form (0.274), trophic guild (0.272), caudal fin form (0.257), and the fourth axis (PCoA4) was mainly correlated with trophic guild (0.429) and body form (0.305).

**FIGURE 3:**
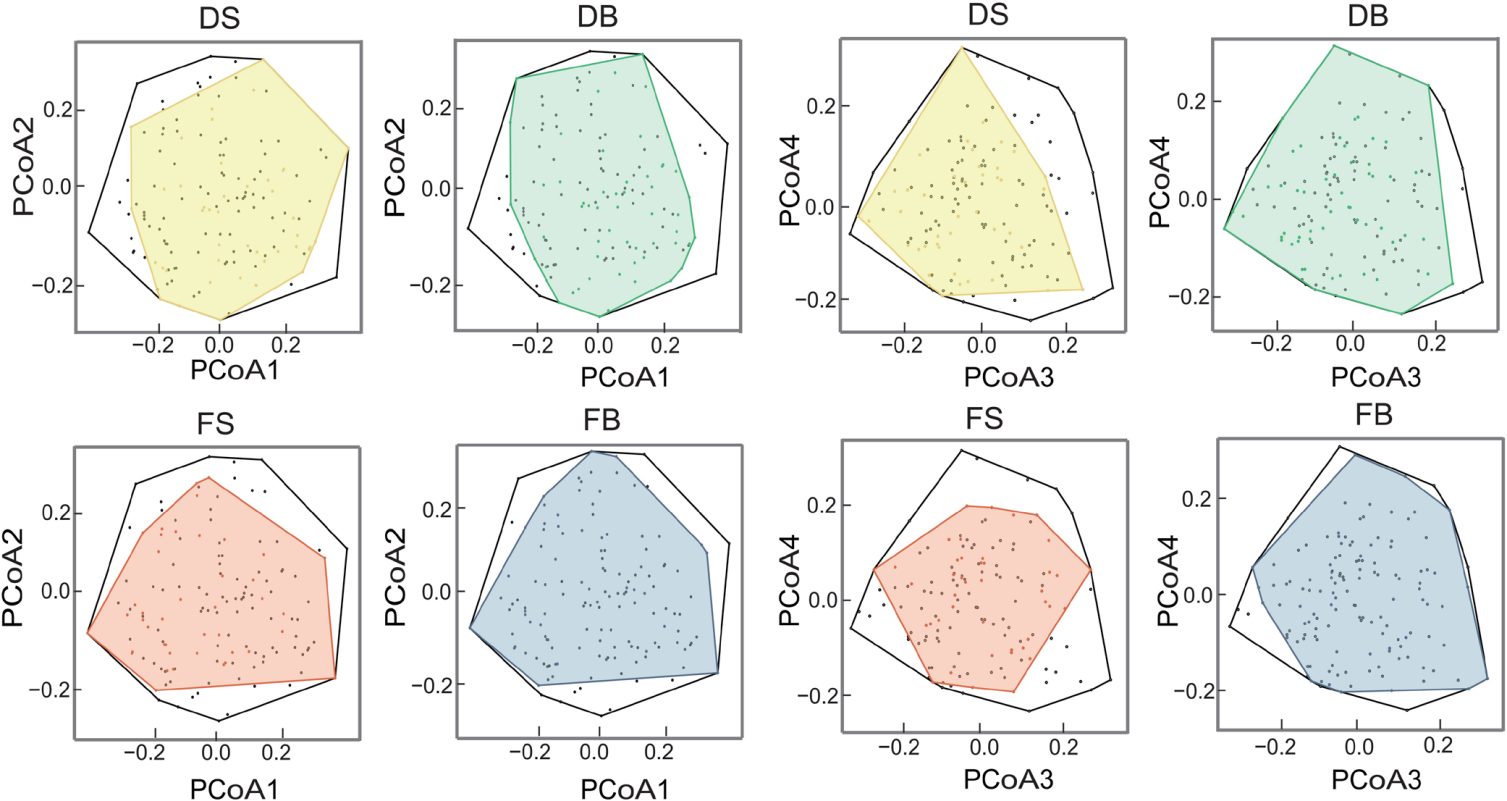
Result of functional space of different fish communities. The convex hulls illustrated the differences in the multidimensional functional space structure defined by the first four axes (PCoA1–PCoA4, representing the main functional gradients). The outermost convex hull filled with black lines and points represented the functional space of all functional entities. The colored filled areas and points represented the functional volume occupied by each group of fish communities. The black hollow points indicated functional entities that were not present in the community.

The functional diversity of fish communities exhibited significant differences between the dry and flood seasons, as indicated by various indices (e.g., FRic, FDiv, FSpe, FOri, and FNND; Figure S2). In the surface layer, FRic and FDiv showed highly significant seasonal differences (*p* < 0.001), while FSpe and FOri exhibited even stronger differences (*p* < 0.0001). In the bottom layer, both FRic and FOri showed significant seasonal differences (*p* < 0.05). Overall, FRic was significantly higher in the flood season than in the dry season, whereas FOri was significantly higher in the dry season. FDiv, FSpe, FNND, and FMPD also tended to be higher in the dry season, but these seasonal differences were not statistically significant (Figure S2).

### 3.3 Model Convergence (HMSC) and Key Drivers for Fish Community Changes

The average PSRFs for the beta, gamma, and omega parameters were all below 1.1, indicating satisfactory model convergence. The effective sample sizes of the MCMC were close to the number of posterior samples, suggesting low auto‐correlation among the MCMC chains (Figure S3). The discriminatory performance of the fish community assembly model was characterized by a mean Tjur *R* ^2^ of 0.46, RMSE of 0.22, and AUC of 0.96 (Figure S4A). The mean predictive discriminatory performance, assessed through four‐fold cross‐validation, yielded a mean Tjur *R* ^2^ of 0.3, RMSE of 0.28, and AUC of 0.86 (Figure S4B). It indicated that the model fitted the training data better than it generalized to new data. The model retained reasonable explanatory and predictive capabilities, making it suitable for investigating the effects on fish community.

Of the variation explained by the HMSC model, fixed effects accounted for 84.4% and random‐effect components accounted for 15.6% (Figure 4). Among individual predictors, pH accounted for the largest proportion of explained variation (17.8%), followed by salinity (16.5%), phosphate (14.7%), and turbidity (11.6%). Total nitrogen, dissolved oxygen, temperature, habitat, season, and sampling site each accounted for less than 10% of the explained variation. Specific environmental variables exerted distinct influences on individual species in the PRE. The dominant species of *Johnius distinctus* , *Tachysurus sinensis*, *Sardinella melanura* , *Coilia mystus* , *Nuchequula nuchalis*, and *Parargyrops edita* were primarily regulated by pH. Similarly, key economic species including *Ilisha elongata* , *Nemipterus japonicus* , *Coilia mystus* , and *Collichthys lucidus* also showed significant associations with pH. Simultaneously, salinity exerted a pronounced influence on the dominant species of *Trichiurus japonicus* , *Upeneus sulphureus* , *Pennahia anea* , *Photopectoralis bindus*, and *Ambassis gymnocephalus* . The economically important species *Johnius belangerii* was also significantly affected by salinity. Furthermore, the variables of phosphate and turbidity were important drivers for several species; phosphate strongly influenced *Nemipterus japonicus* and *Pennahia anea* while turbidity predominantly affected *Odontamblyopus rubicundus* .

**FIGURE 4:**
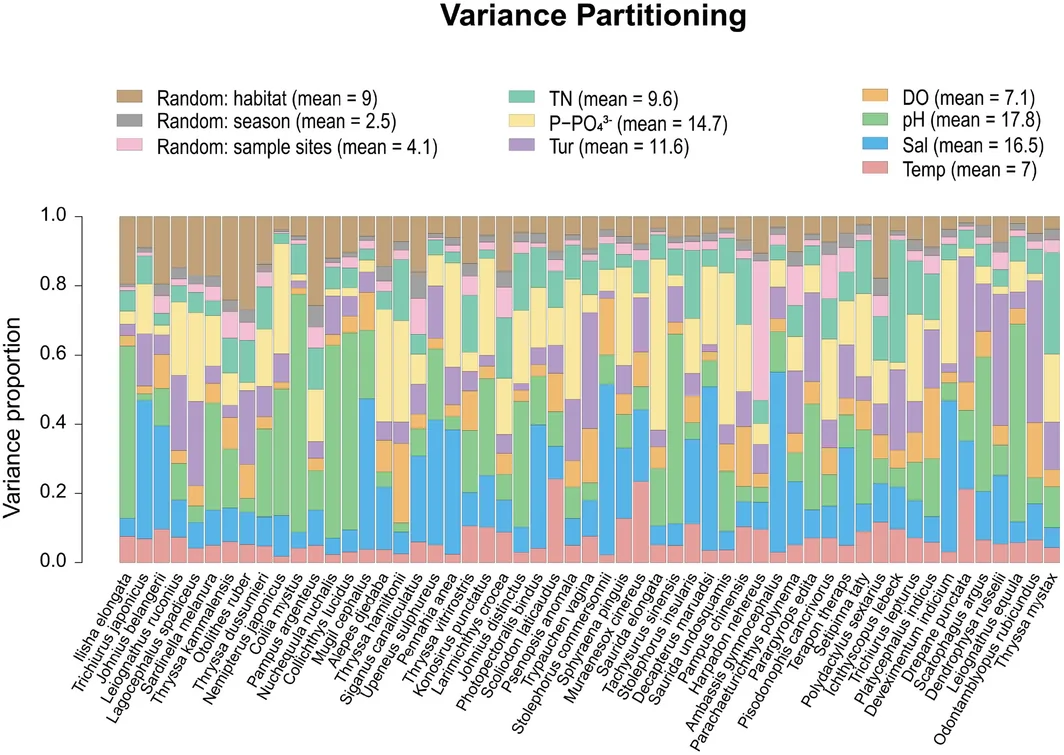
Variance partitioning of the variation in species occurrences explained by the HMSC model. Fixed effects comprised the seven measured environmental covariates, whereas random‐effect components represented sampling site, season, and habitat group. For each species, the stacked bars show the relative contribution of each fixed or random‐effect component to the total variation explained by the model.

### 3.4 Fish Functional Traits‐Environment Relationships

The beta parameters of the model showed that fish species exhibited significant variation in their responses to the environmental variables, indicating pronounced species‐specificity, with responses ranging from significantly positive to significantly negative (Figure S5, posterior probability > 0.9). The beta parameter estimates of the model revealed predominantly positive effects of temperature and total nitrogen for species individual, but negative effects of pH and turbidity for species individual. Moreover, fish functional traits explained 46.3% of the total variance in species occurrences (*R* ^2^_TY_). For different fish functional traits, gamma parameters illustrated the specific response relationship between functional traits and environmental factors (Figure 5). For the continuous traits, body weight and body length exhibited the most frequent associations with environmental factors. Body weight was positively related to salinity, dissolved oxygen, and total nitrogen, but negatively related to pH. In contrast, body length showed negative associations with several environmental variables, including salinity, dissolved oxygen, turbidity, and phosphate. The growth index *K* was negatively related to salinity but positively to pH. Maximum length and temperature preference were negatively associated with temperature, salinity, and phosphate, respectively. Regarding trophic guilds, fish species that feed on zoobenthos showed a negative response to salinity, whereas fish species that feed on plankton exhibited negative responses to both temperature and salinity. In terms of caudal fin form, species with pointed caudal fins were positively associated with dissolved oxygen, turbidity, and total nitrogen, but negatively with pH. Species with truncate or lanceolate caudal fins exhibited positive and negative associations with dissolved oxygen and salinity, respectively. Additionally, across body forms, species with rounded, oval, and flat forms generally showed positive associations with most environmental factors (e.g., temperature, salinity, dissolved oxygen, turbidity, and total nitrogen), whereas flat body form species were negatively related to pH.

**FIGURE 5:**
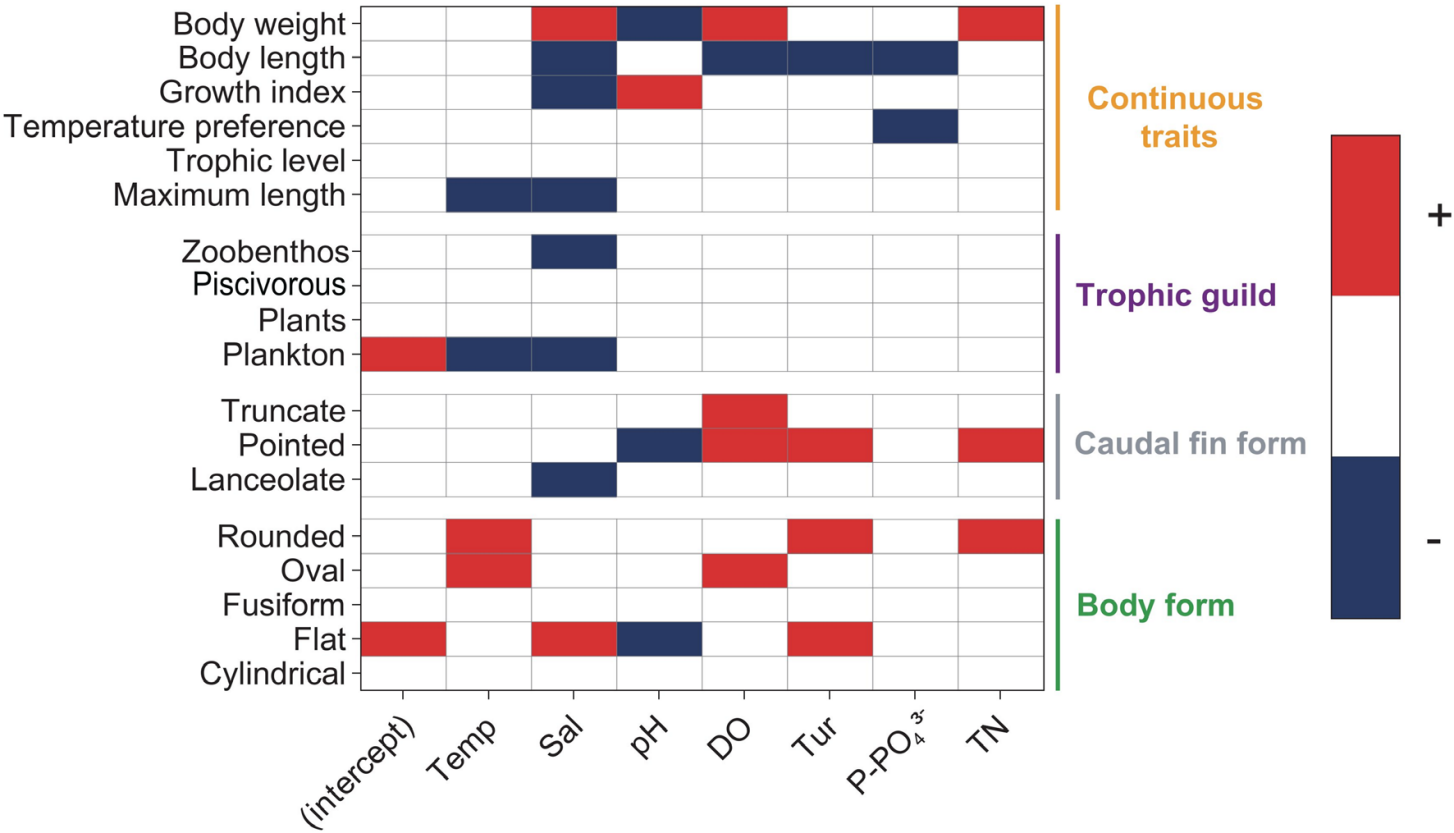
The gamma parameter of the HMSC assessing the relationship between fish functional traits and the environmental covariates, including positive (red), negative (blue), and no relationship (white) (with posterior probability greater than 0.9).

We also quantified the extent to which species traits explained species‐specific responses to environmental factors (*R* ^2^_Tbeta_), with the proportion ranging from 43.5% for turbidity to 72.5% for temperature (Figure S6). Functional traits explained 68.2% of the variation in species responses to phosphate. Therefore, we further examined trait responses along the phosphate gradient and identified both positive and negative associations with phosphate (Figure 6, posterior support > 0.9). Positive associations were observed for growth index *K*, the zoobenthos trophic guild, pointed and truncate caudal fins, and oval and rounded body forms. In contrast, body length, plankton‐ and plant‐feeding trophic guilds, and flat body form showed negative associations with phosphate.

**FIGURE 6:**
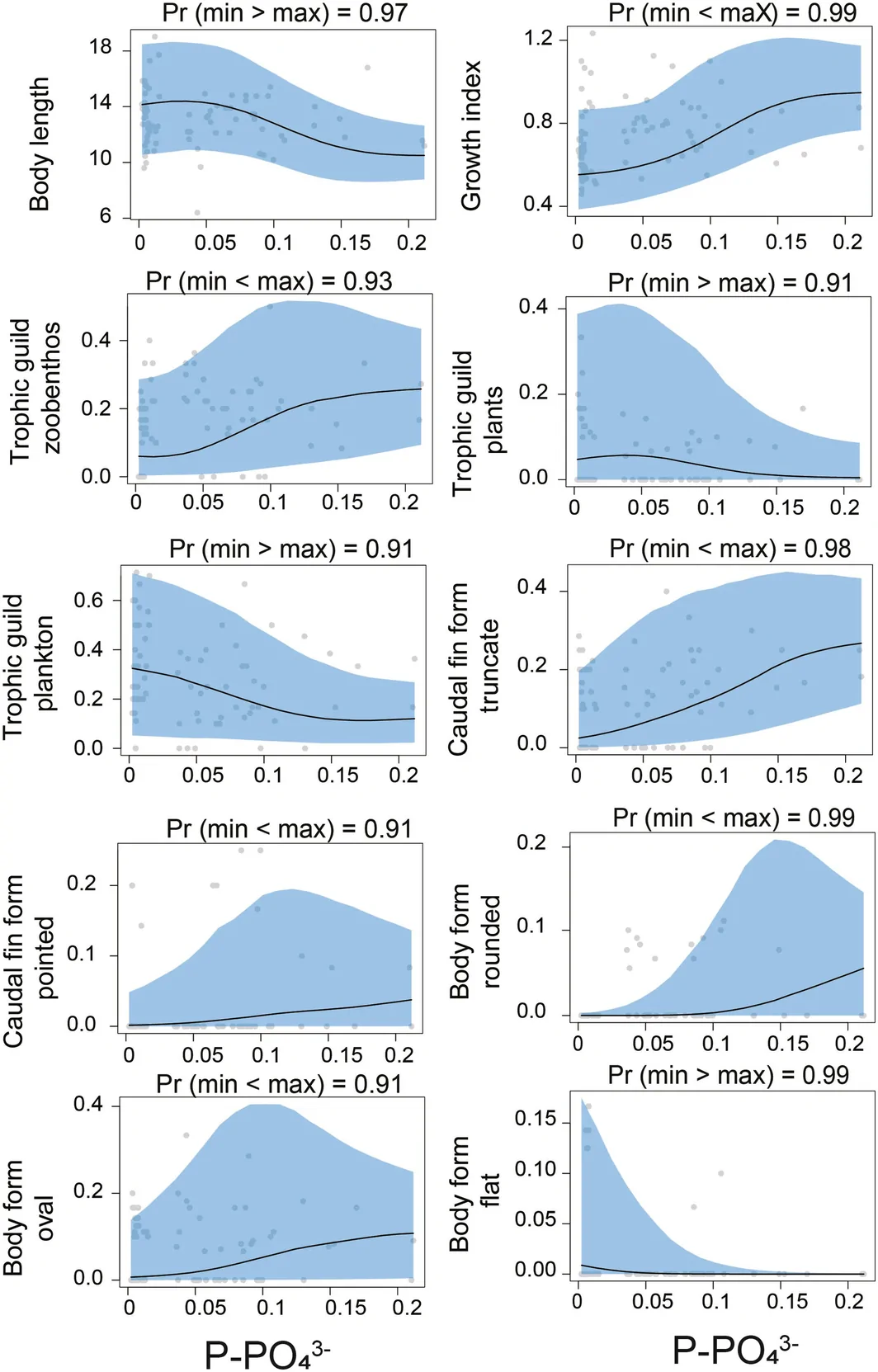
The predicted responses of species traits along the phosphate gradient. The probability value (Pr) represents the posterior probability that the predicted trait value at the maximum phosphate concentration exceeds that at the minimum concentration. A Pr value close to 1 indicates a strong association between the trait and phosphate. Specifically, when the predicted value at the maximum concentration is higher than that at the minimum concentration, it suggests a strong positive association, whereas the opposite pattern indicates a strong negative association. Shaded areas denote 95% credible intervals. Only effects with probabilities greater than 0.90 were reported.

### 3.5 Species Co‐Occurrence

The species residual correlation matrix (the results of omega parameters) associated with the random effect of habitat revealed distinct positive and negative species co‐occurrence patterns (posterior probability > 0.9; Figure 7). For example, dominant surface layer species such as *Ilisha elongata* and *Coilia mystus* exhibited positive associations with other species (e.g., *Johnius belangerii* , *Thryssa hamiltonii* , and *Alepes djedaba* ), but negative associations with several bottom layer species (e.g., *Polydactylus sextarius* , *Trichiurus japonicus* , and *Leiognathus ruconius*). Meanwhile, *Mugil cephalus* showed positive associations with common species including *Pampus argenteus* , *Thryssa dussumieri* , and *Upeneus sulphureus* , but negative associations with *Trichiurus japonicus* and *Leiognathus ruconius*. Similarly, dominant bottom layer species such as *Collichthys lucidus* exhibited positive correlations with other species (e.g., *Leiognathus ruconius* and *Photopectoralis bindus*), but negative associations with several species (e.g., *Mugil cephalus* , *Coilia mystus* , *Pampus argenteus* and *Upeneus sulphureus* ). Likewise, *Odontamblyopus rubicundus* was positively associated with other bottom layer species (e.g., *Polydactylus sextarius* , *Otolithes ruber* , *Platycephalus indicus* ), but negatively associated with multiple pelagic taxa (e.g., *Ilisha elongata* , *Thryssa dussumier* and *Siganus canaliculatus* ).

**FIGURE 7:**
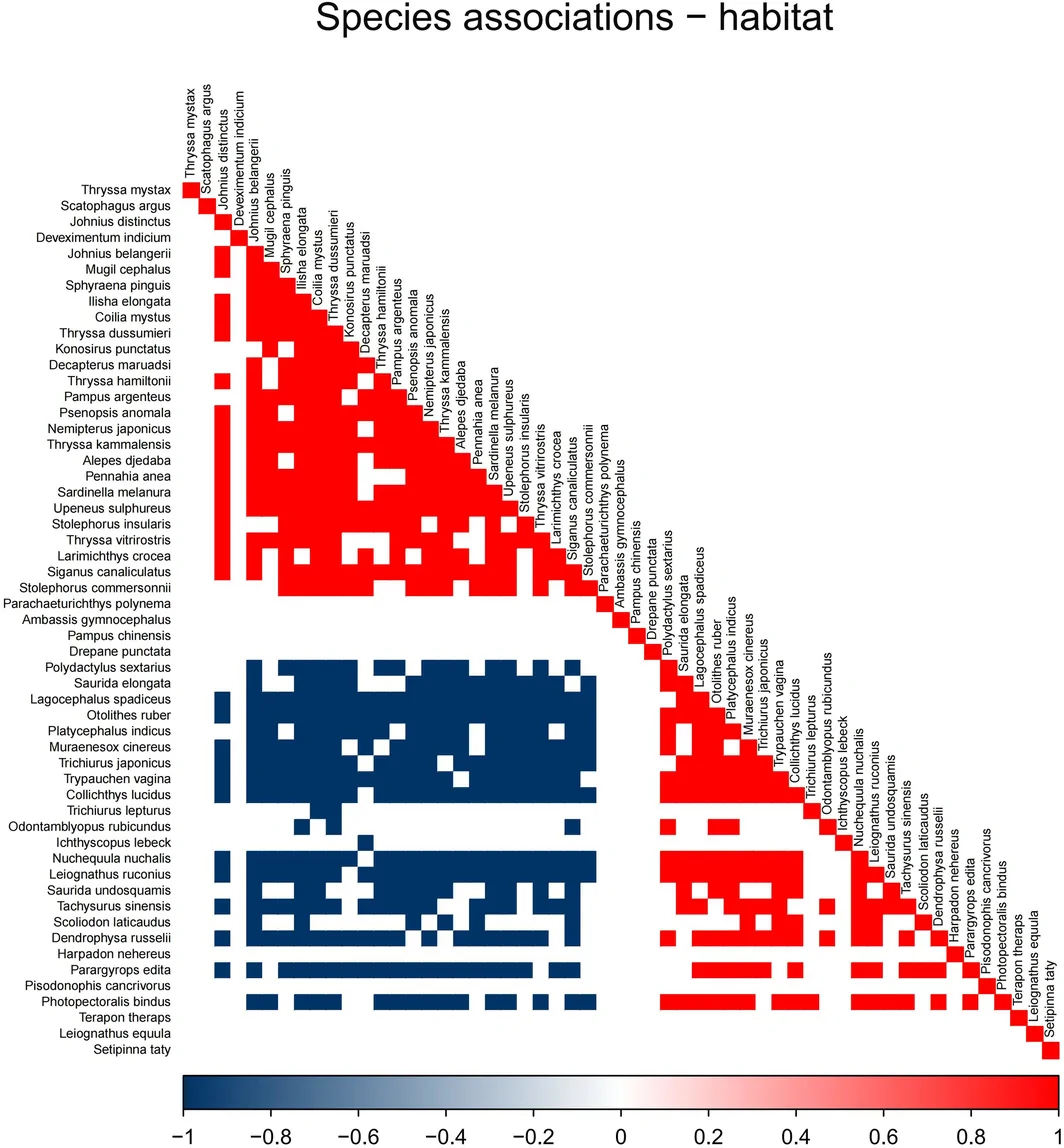
The residual species correlation matrix under the random habitat level. The red and blue colors respectively represented positive and negative correlations between species pairs (the correlation estimation probabilities were both greater than 0.9).

## 4 Discussion

### 4.1 Seasonal Variation in the Functional Traits Composition of Fish Communities

Environmental variables in estuarine ecosystems are highly dynamic across both spatial and temporal scales. Climate‐driven changes in estuarine geography can profoundly influence species distribution patterns, thereby shaping the spatiotemporal dynamics of fish community functional traits (Liu et al. 2023; Rincón‐Díaz et al. 2018; Zhang et al. 2025). The seasonal variation in functional traits reflects the influence of environmental factors, including climate, hydrological conditions, and resource availability, on the ecological characteristics and functional roles of fish communities (Gardmark and Huss 2020; Kuczynski et al. 2018; McKinley et al. 2022; Sutton et al. 2025). In the PRE, fish assemblages exhibited seasonal shifts in functional‐trait composition. Higher values of FRic and FOri indicated that fish communities occupied a broader and more even functional space in the flood season. In contrast, higher FDiv and FSpe suggested greater functional differentiation and specialization among species in the dry season (Figure 3; Figure S2). Furthermore, significantly higher species diversity and FRic in the flood season reflected a more complex community composition and a broader range of functional traits, indicating greater resource availability. It has been demonstrated that overall fish body size tends to increase with the seasonal enhancement of resource availability (Alò et al. 2023; Chea et al. 2020; Pincheira‐Donoso et al. 2019).

Mean body length and body weight tended to be higher in the flood season, with maximum length showing a similar directional pattern (Figure 2A,C,E). It is consistent with the fact that primary productivity in the PRE is typically higher during the flood season, exerting a bottom‐up influence on the seasonal dynamics of fish body size (Lee et al. 2023; Lu and Gan 2015; Tan et al. 2004). According to trophic flow theory, elevated primary productivity can enhance food availability, thereby facilitating ecological interactions and promoting an increase in fish body size (McNab 2010; Norman et al. 2022). In addition, overfishing often leads to resource depletion and community degradation, which can also affect body size structure (Akpalu and Bitew 2014; Lauck et al. 1998). During the fishing moratorium, reduced fishing pressure allows large‐bodied fish to grow and reproduce more successfully, giving them a competitive advantage over smaller species (Castro‐Perez et al. 2025; Xu et al. 2022). The sampling time in our study was conducted in February (dry season, before the fishing moratorium) and September (flood season, after the moratorium). Therefore, the fishing moratorium may have contributed to short‐term increases in fish biomass and body size observed during the flood season.

In terms of trophic guild, fish in the dry season primarily fed on plankton, whereas those in the flood season exhibited a broader diet encompassing plankton, invertebrates, and benthic organisms (Figure 2B). The composition of dominant species across seasons further supported this pattern: planktivorous species such as *Leiognathus ruconius*, *Nuchequula nuchalis*, and *Thrissa vitrirostris* dominated the fish community during the dry season (Figure 1). Previous studies have proved that many estuarine fish species in the PRE enter their spawning or reproductive peaks at the beginning of the dry season, during which their larvae and juveniles rely heavily on plankton as their primary food source (Hou et al. 2021; Wang et al. 2023). Therefore, the higher proportion of planktivorous fish observed in the dry season may reflect the influence of early life history processes on the trophic structure of fish communities. In contrast, the greater trophic diversity of fish communities during the flood season may result from increased nutrient input associated with heavy rainfall, which enhances the productivity of other biological groups and broadens the range of available food resources (Zhang et al. 2022).

### 4.2 Functional Responses of Fish Communities to Environmental Factors

Trait‐based approaches have proven effective in disentangling the complexity and underlying mechanisms of community responses to environmental factors (Diniz et al. 2021; Funk et al. 2023; McLean, Mouillot, Goascoz, et al. 2019; Shivaprakash et al. 2018). In the present study, environmental filtering is the primary factor driving the spatiotemporal differentiation of fish communities in the PRE. The results of HMSC showed higher explanatory than cross‐validated predictive performance (AUC: 0.96/0.86; Tjur's *R* ^2^: 0.46/0.30; RMSE: 0.22/0.28), indicating a certain degree of model optimism (Tikhonov et al. 2020). Nevertheless, the relatively high cross‐validated AUC suggests that the model retained substantial predictive discrimination (Abrego and Ovaskainen 2023). Besides, the predominance of fixed effects in the explained variation (84.4%) is consistent with the strong environmental gradients of the PRE, particularly in pH, salinity, phosphate, and turbidity.

The HMSC gamma parameters further revealed trait‐specific positive and negative responses involving body size, trophic guild, caudal fin form, and body form across multiple environmental gradients (Figures 4 and 5). Higher temperature typically raises individual metabolic and growth rates, thereby increasing energy demands and altering feeding behavior (Li, Guo, et al. 2023). Due to the regional geographic and climatic characteristics, the fish community in the PRE is dominated by warm‐water and warm‐temperate species (Zhou et al. 2019). Functional traits of a general preference for higher temperatures have been revealed in our results (Figure 2D and Figure S5). Consistent with the thermal preference, the proportion of warm‐temperate species was higher during the flood season than the dry season, such as *Photopectoralis bindus*, *Ilisha elongata* , *Collichthys lucidus* , and *Leiognathus equulus* (Figure 1B). The seasonal shift in fish community composition aligned with changes in key functional traits. Besides, the results of CWM and HMSC provide complementary but distinct perspectives. CWM patterns describe abundance‐weighted seasonal changes in the realized trait composition of the fish communities, whereas the HMSC gamma parameters indicate whether particular traits mediate species‐specific occurrence responses to environmental gradients.

The PRE is a fast‐flowing and well‐mixed aquatic ecosystem. Fish species with oval body form and pointed or truncate caudal fin forms achieve higher swimming efficiency and burst acceleration in such ecosystems, enabling them to sustain rapid movement and effectively capture prey, and thus gaining ecological advantages (Leavy and Bonner 2009; Schakmann and Korsmeyer 2023). Our results showed that certain dominant species (e.g., *Ambassis gymnocephalus* , *Clupanodon thrissa* , *Ilisha elongata* , *Odontamblyopus rubicundus* , *Otolithes ruber* , and *Parargyrops edita* ) shared a common pattern of functional trait, characterized notably by an oval body form and pointed or truncated caudal fin forms. Many of the above species are hypoxia‐sensitive, a characteristic frequently linked to their high‐activity lifestyles involving sustained swimming and active foraging, which demand elevated metabolic oxygen (Froese and Pauly 2025; Rogers et al. 2016). The HMSC identified positive associations between dissolved oxygen and several locomotion‐related functional traits, including oval body form and pointed and truncate caudal fin forms (Figure 5; posterior support > 0.9), suggesting that species possessing these traits were more strongly associated with higher dissolved oxygen conditions in the PRE. Besides, the fish with body and fin traits linking to high locomotor performance confer enhanced adaptive advantages in turbid, nutrient rich environments (Bensing et al. 2022). And the positive association between morphology traits and turbidity/total nitrogen likely reflects a productivity‐visibility trade‐off in such environments. Increased turbidity and total nitrogen levels generally enhance the productivity in an aquatic ecosystem but reducing water transparency (Rodrigues et al. 2023). As visual predation efficiency declines, for instance, with rising turbidity, fish may compensate by expanding their movement range, improving maneuverability, or relying more on nonvisual cues (e.g., tactile or hydrodynamic senses) to maintain feeding efficiency (Ranaker et al. 2012; Utne‐Palm 2002).

Although environmental filtering accounted for the dominant component of the modeled fish community variation, residual species associations indicated that it did not fully account for species co‐occurrence patterns in the PRE (Figure 7). Both positive and negative associations remained after accounting for the measured environmental covariates, potentially reflecting shared habitat or resource preferences, life‐history synchrony, habitat partitioning, biotic interactions, or responses to unmeasured environmental factors (Ovaskainen et al. 2017; Blanchet et al. 2020; Pichler and Hartig 2021). For example, positive associations among *Coilia mystus* , *Johnius belangerii* , and *Thryssa hamiltonii* may reflect similar habitat requirements or seasonal life‐history dynamics, whereas negative associations among species occupying different ecological layers may partly reflect habitat partitioning (Zhang et al. 2021; Zeng et al. 2022). These residual associations should not be interpreted as direct evidence of species interactions because HMSC cannot distinguish biotic effects from unmeasured environmental or spatial processes (Pichler and Hartig 2021). Nevertheless, they indicate that additional ecological processes may contribute to fish community assembly in the PRE.

### 4.3 Key Drivers of Salinity and Phosphate for Fish Functional Traits

Consistent with the commonly observed trait‐environment coupling pattern in marine fishes, both the Growth index K and maximum length were negatively correlated with salinity (Beukhof et al. 2019). It may be attributed to the increased energetic cost of osmoregulation under high salinity conditions, which elevates physiological stress and metabolic load, thereby constraining growth potential (Zikos et al. 2014). Interestingly, body length showed a negative correlation with salinity, but body weight exhibited a positive one, suggesting that these two traits may represent distinct ecological strategies and respond differently to the same environmental driver. Such “decoupled ecological responses” have been previously documented (Laughlin 2014; Loi et al. 2022; Winemiller et al. 2015; Zikos et al. 2014). Besides, trophic guild traits associated with feeding on plankton and zoobenthos were negatively correlated with salinity, whereas flat body form fish showed a positive correlation. This pattern may result from the relatively narrow salinity tolerance of many zoobenthos, as salinity fluctuations can alter sediment chemistry and benthic habitats, reducing the availability of food resources for benthivorous species (Able 2005; Barletta et al. 2003). Conversely, flat body form fishes such as *Solea ovata* and *Cynoglossus puncticeps* tend to prefer coastal or estuarine habitats with sandy or muddy substrates and relatively high salinity (Chrisp et al. 2023). To sum up, salinity influences fish community by affecting fish energy utilization strategies and trophic guild, thereby shaping the distribution of multiple functional traits.

Notably, our results revealed that phosphate explained 14.7% of the variation in species distribution, with functional traits accounting for 68.2% of species' responses to phosphate, indicating that phosphate is a key environmental factor shaping fish community distribution patterns in the PRE, and different functional traits contributed unequally to species' responses to phosphate (Figure 4 and Figure S6). The HMSC trait‐response predictions identified positive associations of phosphate with the von Bertalanffy growth coefficient *K*, the zoobenthos‐feeding guild, pointed and truncate caudal fins, and oval and rounded body forms. In contrast, body length, plankton‐ and plant‐feeding guilds, and flat body form showed negative associations with phosphate (Figure 6; posterior support > 0.90). This may be attributed to the fact that elevated phosphate levels enhance primary productivity, promoting the proliferation of plankton and small prey organisms and thereby increasing food availability (Wang 2024). And fish species with highly maneuverable caudal fin forms (e.g., *Ichthyscopus lebeck* in our results) likely gain greater advantages in foraging efficiency and energy utilization in such highly productive environments, leading to their increased dominance in the community (Cermeño et al. 2016; Norman et al. 2022). Conversely, plankton trophic guild were negatively correlated with phosphate. This pattern may be linked to the ecological preferences of pelagic or migratory species (e.g., *Trichiurus lepturus* in our results), which typically inhabit offshore or open‐water environments characterized by greater stability and lower turbidity (Gleiber et al. 2024; Seers and Shears 2015). Thus, phosphate could regulate primary productivity, indirectly determining the distribution of body form and caudal fin form traits associated with locomotor performance. Although prevalence filtering was necessary to improve model stability, several infrequently detected species of conservation or fisheries importance were excluded from the HMSC analysis. Consequently, the HMSC‐based inference primarily represents the environmental responses of relatively widespread species and may not fully capture the environmental sensitivity of rare taxa.

## 5 Conclusions

Elucidating how fish communities respond to environmental variations is essential for the sustainable development of estuarine ecosystems and remains a key question in estuarine ecology. HMSC provides a comprehensive framework for exploring these response mechanisms by jointly modeling species occurrences, environmental covariates, species functional traits, and spatiotemporal random effects. In the study, we investigated seasonal variations in fish community composition, taxonomic diversity, functional diversity, and the environmental drivers in the PRE. Our results revealed pronounced seasonal differences in fish community composition, taxonomic diversity, functional diversity, and functional space occupation. Seasonal shifts were observed in several CWM traits, including body size, temperature preference, growth, and trophic‐guild composition, yet without statistically significant differences. Furthermore, environmental variables accounted for the majority of the explained variation in the HMSC model, indicating environmental filtering emerged as the primary process shaping fish community distribution patterns. Compared with other environmental factors, pH, salinity and phosphate exerted stronger influences on both species composition (e.g., *Photopectoralis bindus*, *Trichiurus japonicus* , and *Pennahia anea* ) and functional traits (including body length, body weight, body form, and caudal fin form) of fish communities, suggesting that the future changes in these three factors may substantially affect fish community dynamics in the PRE. Overall, these findings enhance our understanding of how fish communities respond to environmental change and underscore the critical role of functional traits in these responses, thereby laying an important foundation for developing effective management and conservation strategies for fish communities in the future.

## Author Contributions

**Longjie Zhang:** data curation (equal), investigation (equal), visualization (equal). **Wencheng Zhao:** conceptualization (equal), investigation (equal), visualization (equal), writing – original draft (equal). **Shuai Zhang:** funding acquisition (equal), supervision (equal). **Shiyi Li:** data curation (equal), methodology (equal), resources (equal). **Min Li:** conceptualization (equal), funding acquisition (equal), project administration (equal). **Keshu Zou:** conceptualization (equal), methodology (equal), supervision (equal), writing – review and editing (equal).

## Funding

This work was supported by the National Key Research and Development Program, China (2024YFD2401402), National Natural Science Foundation of China (32102793), and Guangzhou Science and Technology Program Project (2025A04J4565).

## Disclosure

Declaration of Generative AI and AI‐assisted technologies in the writing process: No generative Al or Al‐assisted technologies were used in the research process or writing of this manuscript.

## Ethics Statement

All animal experiments were approved by the Committee on the Ethics of Animal Experimentation of South China Agricultural University (permit no. 2022C042).

## Conflicts of Interest

The authors declare no conflicts of interest.

## Supporting information


**Figure S1:** Diversity analysis results of fish communities in different groups: (A) α‐diversity results of different fish communities (****, *p* ≤ 0.0001; ***, *p* ≤ 0.001; **, *p* ≤ 0.01; *, *p* ≤ 0.05); (B) NMDS analysis results of different fish communities.

**Figure S2:** Results of functional diversity in different fish communities. Functional diversity indices include Functional Richness (FRic), Functional Divergence (FDiv), Functional Specialization (FSpe), Functional Originality (FOri), Functional Mean Pairwise Distance (FMPD), and Functional Nearest Neighbor Distance (FNND). The statistical significance levels were indicated as follows: **** (*p* ≤ 0.0001); *** (*p* ≤ 0.001); ** (*p* ≤ 0.01); * (*p* ≤ 0.05).

**Figure S3:** Model parameter fitting results evaluated based on the effective sample size (ESS) and the potential scale reduction factor (PSRF). An ESS close to 4000 (the total number of samples per model) indicated low autocorrelation between consecutive samples. A PSRF value < 1.1 suggested good convergence, with consistent results across MCMC chains.

**Figure S4:** Explanatory and predictive performance of the HMSC model based on three evaluation metrics: root mean square error (RMSE), the area under the curve (AUC) and Tjur *R* ^2^. (A) Model fit to training data; (B) Predictive performance under 4‐fold cross‐validation. AUC reflected the model's ability to discriminate between presence and absence, with values of 0.5 indicating random performance, 0.7–0.8 acceptable, 0.8–0.9 excellent, and > 0.9 outstanding. RMSE reflected the average deviation between predicted and observed values; lower RMSE indicated higher predictive accuracy. Tjur *R* ^2^ represents the difference between the mean predicted probabilities for observed presences and observed absences; higher values indicate greater discriminatory ability.

**Figure S5:** The beta parameters of the HMSC used to assess the responses of fish species to environmental covariates, including positive (red), negative (blue), or no relationship (blank) (posterior probability greater than 0.9).

**Figure S6:** The proportion of variation in species responses to environmental factors explained by traits (*R* ^2^_Tbeta_).

**Table S1:** Functional traits used to characterize the fish community in this study. The traits selected represented key aspects of fish adaptability to estuarine environments and were instrumental in analyzing functional diversity and understanding community assembly processes. While not exhaustive, the chosen traits were widely recognized as ecologically relevant for estuarine fish species. All traits were coded as either continuous (numerical) or categorical (factor) variables. Most trait information was sourced from FishBase, with missing values supplemented using data from closely related taxa or relevant literature. Measurements of body length and weight were derived from averages of the sampled individuals.
**Table S2:** Mean absolute deviation (MAD) values across the first 10 axes (PCoA1‐PCoA10) of the functional space constructed for different fish communities. DB, FB, DS, and FS respectively represented different fish communities. Lower MAD values indicated higher stability of the functional space along a given axis, reflecting a tighter clustering of species.
**Table S3:** Seasonal comparisons of fish functional traits and trophic‐guild composition in surface and bottom‐associated communities. Both unadjusted and Benjamini‐Hochberg‐adjusted *p* values are reported.
**Table S4:** Correlations between species traits and the first seven functional axes PCoA1 to PCoA7, which represented the major dimensions of the functional space. Values indicated the correlation coefficients between trait values and axis scores, with significance levels denoted as **** (*p* ≤ 0.0001); *** (*p* ≤ 0.001); ** (*p* ≤ 0.01); * (*p* ≤ 0.05).

## Data Availability

All data and R code supporting this study have been deposited in the Dryad Digital Repository (https://doi.org/10.5061/dryad.cnp5hqcn2). The files will become publicly accessible following completion of the Dryad curation process and are released under the Creative Commons Zero v1.0 Universal (CC0 1.0) Public Domain Dedication.
