# Supplementary material for: Disentangling the Drivers of Fish Community Variation in a Highly Urbanized Estuary Based on Hierarchical Modeling of Species Communities (HMSC)
Source: Ecol Evol. 2026 Oct 26;16(10):e74523. doi: 10.1002/ece3.74523 (PMC13710892; doi:10.1002/ece3.74523)
Supplement: Supplementary file 1 [file ECE3-16-e74523-s002.docx]

Table S1 Functional traits used to characterize the fish community in this study. The traits selected represented key aspects of fish adaptability to estuarine environments and were instrumental in analyzing functional diversity and understanding community assembly processes. While not exhaustive, the chosen traits were widely recognized as ecologically relevant for estuarine fish species. All traits were coded as either continuous (numerical) or categorical (factor) variables. Most trait information was sourced from FishBase, with missing values supplemented using data from closely related taxa or relevant literature. Measurements of body length and weight were derived from averages of the sampled individuals.

| Category | Functional traits | Type | Units or categories |
| --- | --- | --- | --- |
| Morphology | Body form | Categorical | Compressed; Oval; Fusiform; Rounded; Flat; Cylindrical |
|  | Caudal fin form | Categorical | Forked; Heterocercal; Pointed  Truncate |
| Life history | Maximum length | Continuous | cm |
|  | Body length | Continuous | cm |
|  | Body weight | Continuous | g |
|  | Von Bertalanffy growth coefficient (K) | Continuous | year^-1^ |
| Nutritional ecology | Trophic guild | Categorical | Plankton; Invertebrates;  Piscivorous; Zoobenthos |
|  | Trophic level | Continuous | - |
| Habitat use | Temperature preference | Continuous | ℃ |

Table S2 Mean absolute deviation (MAD) values across the first ten axes (PCoA1-PCoA10) of the functional space constructed for different fish communities. DB, FB, DS, and FS respectively represented different fish communities. Lower MAD values indicated higher stability of the functional space along a given axis, reflecting a tighter clustering of species.

| Mad | DB | FB | DS | FS |
| --- | --- | --- | --- | --- |
| PCoA1 | 0.26 | 0.249 | 0.22 | 0.241 |
| PCoA2 | 0.167 | 0.164 | 0.134 | 0.149 |
| PCoA3 | 0.111 | 0.11 | 0.091 | 0.098 |
| PCoA4 | 0.073 | 0.073 | 0.063 | 0.065 |
| PCoA5 | 0.051 | 0.052 | 0.048 | 0.048 |
| PCoA6 | 0.04 | 0.04 | 0.036 | 0.04 |
| PCoA7 | 0.035 | 0.039 | 0.034 | 0.038 |
| PCoA8 | 0.039 | 0.041 | 0.037 | 0.041 |
| PCoA9 | 0.045 | 0.049 | 0.043 | 0.048 |
| PCoA10 | 0.05 | 0.057 | 0.047 | 0.053 |

Table S3 Seasonal comparisons of fish functional traits and trophic-guild composition in surface and bottom-associated communities. Both unadjusted and Benjamini-Hochberg-adjusted *P* values are reported.

| Comparison | Trait | Test | Direction / summary | Raw *P* | BH-adjusted *P* |
| --- | --- | --- | --- | --- | --- |
| DS vs FS | Maximum length | Paired permutation | Flood > Dry | 0.0566 | 0.08136 |
| DS vs FS | Trophic level | Paired permutation | Flood > Dry | 0.4888 | 0.4888 |
| DS vs FS | Temperature preference | Paired permutation | Flood < Dry | 0.0467 | 0.08136 |
| DS vs FS | Growth coefficient K | Paired permutation | Flood > Dry | 0.0093 | 0.0558 |
| DS vs FS | Length | Paired permutation | Flood > Dry | 0.0359 | 0.08136 |
| DS vs FS | weight | Paired permutation | Flood > Dry | 0.0678 | 0.08136 |
| DB vs FB | Maximum length | Paired permutation | Flood < Dry | 0.5179 | 0.77685 |
| DB vs FB | Trophic level | Paired permutation | Flood > Dry | 0.151 | 0.453 |
| DB vs FB | Temperature preference | Paired permutation | Flood > Dry | 0.0954 | 0.453 |
| DB vs FB | Growth coefficient K | Paired permutation | Flood < Dry | 0.4226 | 0.77685 |
| DB vs FB | Length | Paired permutation | Flood > Dry | 0.7621 | 0.8376 |
| DB vs FB | weight | Paired permutation | Flood > Dry | 0.8376 | 0.8376 |
| DS vs FS | Body.shape | Stuart–Maxwell | composition differed | 0.124064 | 0.186096 |
| DS vs FS | Caudal fin shape | Stuart–Maxwell | composition differed | 0.572407 | 0.572407 |
| DS vs FS | Diet | Stuart–Maxwell | composition differed | 0.037111 | 0.111332 |
| DB vs FB | Body shape | Stuart–Maxwell | composition differed | 0.335911 | 0.335911 |
| DB vs FB | Caudal.fin.shape | Stuart–Maxwell | composition differed | 0.261464 | 0.335911 |
| DB vs FB | Diet | Stuart–Maxwell | composition differed | 0.033923 | 0.101769 |

Table S4 Correlations between species traits and the first seven functional axes PCoA1 to PCoA7, which represented the major dimensions of the functional space. Values indicated the correlation coefficients between trait values and axis scores, with significance levels denoted as **** (*P* ≤ 0.0001); *** (*P* ≤ 0.001); ** (*P* ≤ 0.01); * (*P* ≤ 0.05).

| Traits | PCoA1 | PCoA2 | PCoA3 | PCoA4 | PCoA5 | PCoA6 | PCoA7 |
| --- | --- | --- | --- | --- | --- | --- | --- |
| Body form | 0.104** | 0.507**** | 0.274**** | 0.305**** | 0.144*** | 0.131*** | 0.059* |
| Caudal fin form | 0.343**** | 0.379**** | 0.257**** | 0.08* | 0.261**** | 0.051 | 0.071* |
| Maximum length | 0.404* | 0.09 | 0.097 | 0.092 | 0.058 | 0.138 | 0.049 |
| Trophic guild | 0.293**** | 0.105** | 0.272**** | 0.429**** | 0.326**** | 0.241**** | 0.66**** |
| Trophic level | 0.324 | 0.09 | -0.027 | 0.027 | -0.089 | 0.047 | -0.088 |
| Temperature preference | 0.11 | -0.08 | -0.013 | -0.022 | 0.115 | 0.267* | 0.197* |
| Growth index K | 0.313**** | 0 | 0.001 | 0.009 | 0.008 | 0 | 0 |
| Body length | 0.526* | 0.159 | 0.26 | 0.2 | -0.076 | -0.11 | -0.222 |
| Body weight | 0.617 | -0.528 | 0.516 | 0.341 | -0.07 | 0.301 | 0.213 |
